# Fungal co-infection in COVID-19 patients: evidence from a systematic review and meta-analysis

**DOI:** 10.18632/aging.202742

**Published:** 2021-03-19

**Authors:** Jingwen Peng, Qiong Wang, Huan Mei, Hailin Zheng, Guangzhao Liang, Xiaodong She, Weida Liu

**Affiliations:** 1 Department of Medical Mycology, Institute of Dermatology, Chinese Academy of Medical Science and Peking Union Medical College, Nanjing 210042, Jiangsu, China; 2 Jiangsu Key Laboratory of Molecular Biology for Skin Diseases and STIs, Nanjing 210042, Jiangsu, China; 3 Center for Global Health, School of Public Health, Nanjing Medical University, Nanjing 211166, China

**Keywords:** fungal co-infection, COVID-19, meta-analysis, systematic review

## Abstract

Coronavirus disease 2019 (COVID-19) has infected tens of millions of people worldwide within the last year. However, the incidence of fungal co-infection in COVID-19 patients remains unclear. To investigate the association between fungal co-infection and mortality due to COVID-19, we systematically searched Medline, Embase, MedRxiv and Cochrane Library for eligible studies published in the period from 1 January to 1 December 2020. We performed a meta-analysis of nine studies that met the inclusion criteria. In total, data from 2780 patients and 426 patients were included who were admitted to the ICU. In eight of the articles, 211 participants died due to COVID-19 infection, which means an overall mortality rate of 10.9%. The overall pooled proportion of fungal co-infection in COVID-19 patients was 0.12 (95% CI = 0.07-0.16, n = 2780, *I*^2^ = 96.8%). In terms of mortality in COVID-19 patients with fungal infection, the overall pooled proportion of mortality was 0.17 (95% CI = 0.10-0.24, n = 1944, *I*^2^ = 95.6%). These findings provide evidence suggesting a favorable use for empirical antibiotics in the majority of patients when COVID-19 infection is diagnosed. Our analysis is investigating the use of antifungal therapy to treat COVID-19 can serve as a comprehensive reference for COVID-19 treatment.

## INTRODUCTION

The recent outbreak of coronavirus disease 2019 (COVID-19), a new disease mostly manifested as viral pneumonia, started as a local epidemic but developed within a few months into a worldwide pandemic with high morbidity and mortality rates [1–3]. However, breakthroughs in the development of specific therapeutic agents are still needed and newly developed vaccines against COVID-19 infection are available only in small quantity. Thus, the most effective way to address the pandemic is the prevention of further infection. This is achieved by using specific strategies such as early diagnosis and subsequent quarantine, and policies preventing gatherings, such as enforced social distancing. From the previous coronavirus outbreak of severe acute respiratory syndrome (SARS), we found that fungal co-infection of coronavirus patients could significantly increase mortality rates [4–7]. The significance of fungal co-infection in COVID-19 patients, however, especially in patients with severe and critical conditions, is still poorly understood.

As with other respiratory diseases such as influenza, where approximately 25% of elderly patients acquire secondary co-infections, a similar co-infection has also been seen in COVID-19 patients [6]. However, data remain limited regarding the impact of fungal co-infection and associated clinical outcomes. In many studies looking at the treatment of COVID-19 patients, the empirical use of antibiotics for the majority of patients is known [6–9]. However, there is evidence that inflammatory serological markers, which are usually associated with bacterial infection, such as raised procalcitonin and C-reactive protein, may appear in patients with COVID-19 who have no bacterial co-infection. Paradoxically, the administration of antibiotics prevents co-infection, which has been associated with a more severe clinical condition in COVID-19 patients [10–12]. Therefore, there is a clinical demand for a robust investigation into the role of co-infection in patients with COVID-19.

In this study, we performed a systematic review and meta-analysis of nine studies investigating fungal co-infection in COVID-19 patients, to estimate the association between fungal co-infection and mortality. These findings may help to inform future clinical management and treatment of COVID-19 patients. In the context of rising levels of antimicrobial resistance, we aim to enable a sustainable and judicious antibiotic administration.

## RESULTS

### Study selection and characteristics

**Figure 1:**
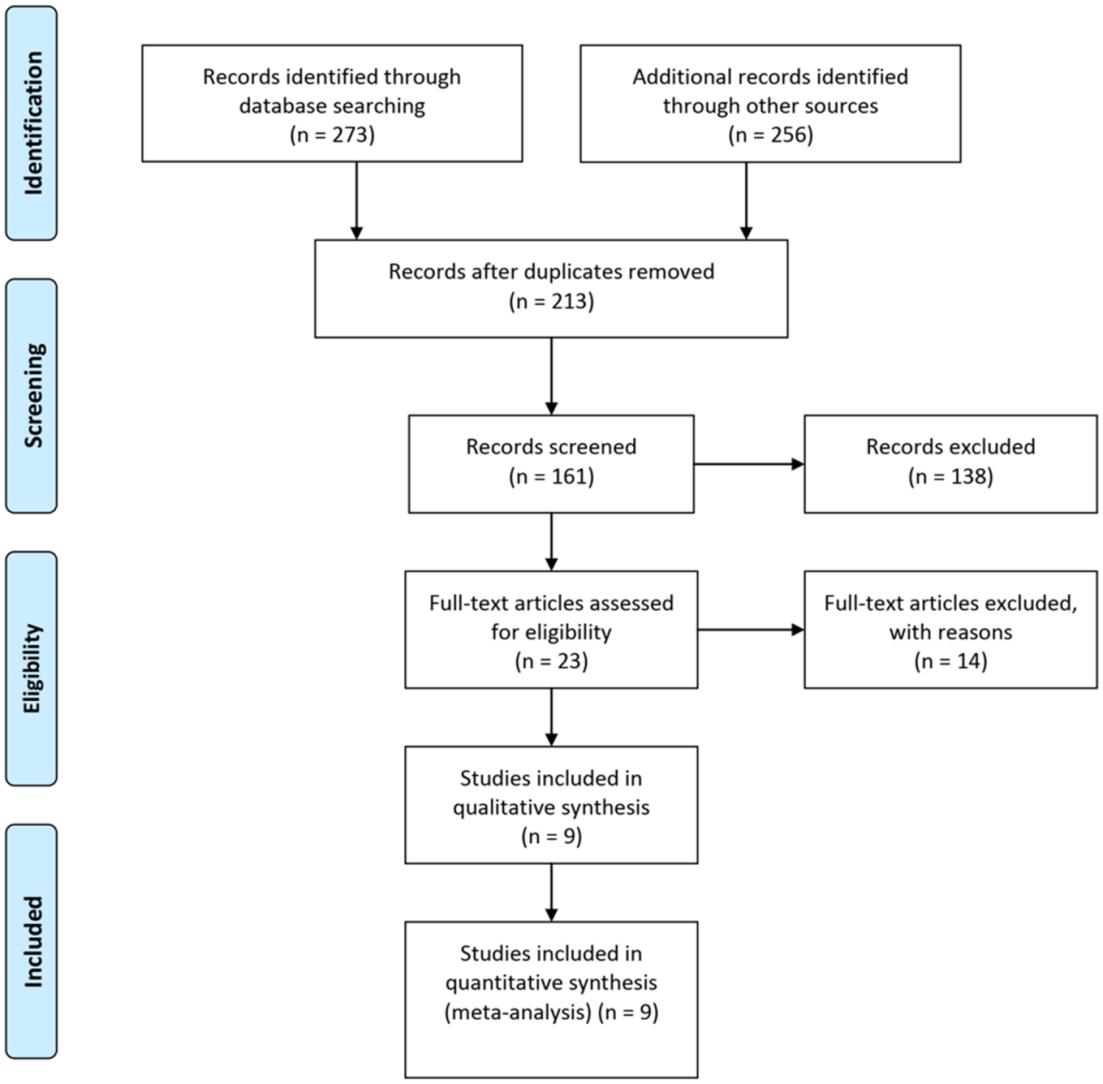
**Flow diagram of the study selection process.**

The combined search terms yielded 273 articles and a primary review of the titles and abstracts identified 32 articles that warranted a full manuscript review. After screening the literature based on the inclusion criteria, nine articles were identified as potentially relevant articles (Figure 1). In total, nine studies including 2780 participants and 426 patients admitted to the ICU met the inclusion criteria and were selected for meta-analysis. Of the participants, there was 211 death reported in eight of the articles, for an overall mortality rate of 10.9%. In one article, the outcomes were not reported. Four (44%) of the included studies were from China, two were from the United Kingdom (22%), one (11%) was from Pakistan, and one (11%) was from Spain. The characteristics of the selected studies are summarized in Table 1.

**Table 1:** Characteristics of all studies describing fungal co-infections in the meta-analysis.

**Author**	**Country**	**Race**	**Severity**	**Study type**	**Method**	**Total**	**ICU**	**Deaths (%)**	**Fungal (%)**	**Aspergillus (%)**
Yang [5]	China	Asian	ICU	Case series	Culture	52	52	32(61.5%)	3(5.8%)	2(3.8%)
Wang [13]	China	Asian	Mix	Case series	Culture	29		2(6.9%)	2(6.9%)	0(0%)
Chen [19]	China	Asian	ICU	Case series	Culture	99	23	11(11.1%)	4(4.0%)	1(1.0%)
Zhu [10]	China	Asian	Mix	Retrospective study	RT-PCR	257	3	0(0%)	60(23.3%)	66(25.7%)
White [11]	UK	Caucasian	ICU	Prospective study	Mix	135	135	51(37.8%)	36(26.7%)	19(14.1%)
Hughes [7]	UK	Caucasian	Mix	Retrospective study	Mix	836			27(3.2%)	3(0.4%)
Nasir [6]	Pakistan	Caucasian	Mix	Retrospective study	Culture	147	23	4(2.7%)	9(6.1%)	9(6.1%)
Garcia-Vidal [16]	Spain	Caucasian	Mix	Prospective study	Culture	989	146	97(9.8%)	7(0.7%)	3(0.3%)

Mix of Severity, Symptoms of the disease include moderate, severe, and critical; Mix of the Method, Detection methods include culture, serological diagnosis, and reverse transcription-polymerase chain reaction (RT-PCR); ICU, intensive-care unit; UK, United Kingdom.

**Figure 2:**
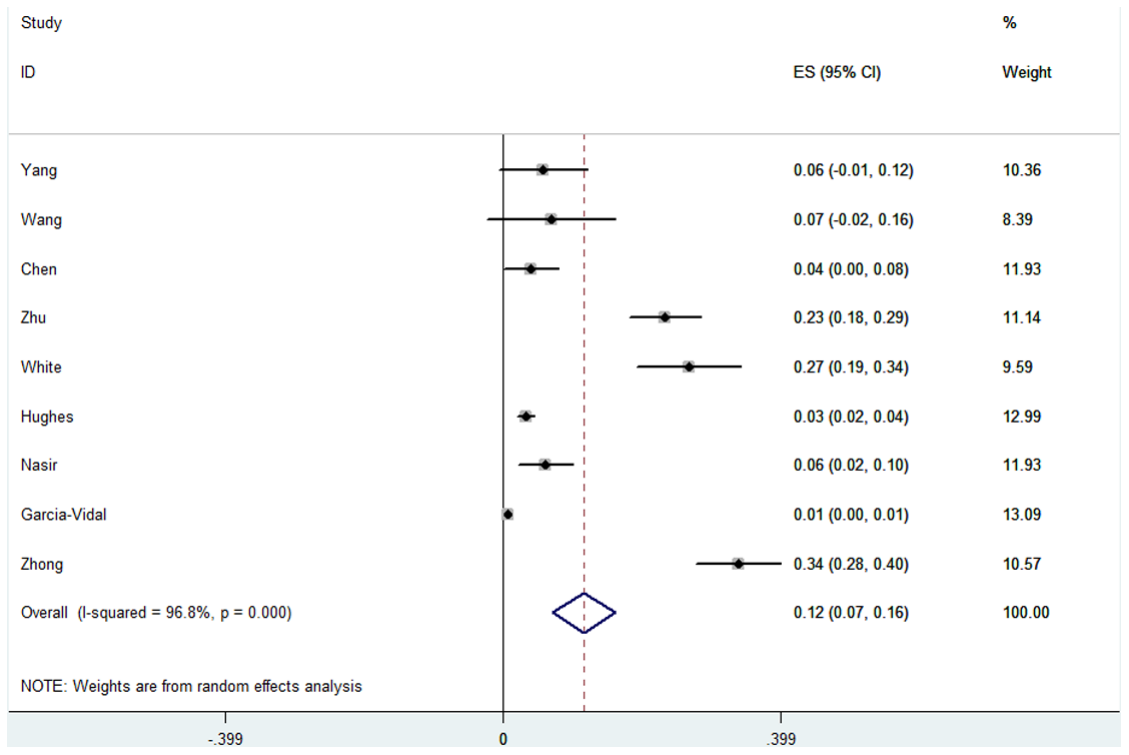
**Forest plot of fungal infection in COVID-19 patients.**

For fungal co-infection in COVID-19 patients, the overall pooled proportion of patients who had laboratory-confirmed fungal co-infection was 0.12 [(95% confidence intervals (CI) = 0.07-0.16, n = 2780, *I*^2^ = 96.8%)]. Subgroup analysis from studies with separate data for Asian patients showed that a greater proportion of them had fungal co-infections when compared to the patients in the studies from the U.K. and Spain (0.15, 95% CI = 0.03-0.27), n = 679, *I*^2^ = 95.5% versus 0.07, 95% CI = 0.03-0.10, n = 2101, *I*^2^ = 95.4%). Subgroup analyses were not significantly different for the severity of disease, study type, or test method. Sensitivity analysis excluding one study, did not significantly affect the overall proportion of patients with fungal co-infection, nor did it decrease the heterogeneity (0.14, 95% CI = 0.06-0.21, *I*^2^ = 96.0%). There were no data from the remaining studies relating to the time from admission to detection of co-infection. Specific data are summarized in Figure 2, Table 2, and Supplementary Figure 1.

**Table 2:** Proportion of fungal co-infections and COVID-19.

	**Proportion(95%CI)**	***P***	***P_h_***	***I*^2^%**
**Total**	0.12(0.07-0.16)	0.000	0.000	96.8%
**Race**				
Asian	0.15(0.03-0.27)	0.015	0.000	95.5%
Caucasian	0.07(0.03-0.10)	0.001	0.000	95.4%
**Severity**				
ICU	0.12(-0.01-0.24)	0.065	0.000	93.0%
MIX	0.12(0.06-0.17)	0.000	0.000	97.5%
**Study type**				
Case series	0.05(0.02-0.08)	0.003	0.804	0.0%
Retrospective study	0.16(0.04-0.29)	0.010	0.000	97.9%
Prospective study	0.13(-0.12-0.39)	0.301	0.000	97.8%
**Method**				
Culture	0.04(0.01-0.07)	0.016	0.008	71.2%
RT-PCR	0.23(0.18-0.29)	0.000	-	-
MIX	0.15(-0.08-0.38)	0.211	0.000	97.3%
Fungus chip	0.34(0.28-0.40)	0.000	-	-
**Case size**				
>200	0.14(0.08-0.20)	0.000	0.000	98.5%
<200	0.10(0.03-0.16)	0.003	0.804	0.0%

Mix of Severity, Symptoms of the disease include moderate, severe and critical; Mix of the Method, Detection methods include culture, serological diagnosis, reverse transcription-polymerase chain reaction; (RT-PCR); ICU, intensive-care unit; CI, confidence interval; *P*_h_, *P* value of heterogeneity, *P* value of Q-test for the heterogeneity test; *I*^2^, 0–25, no heterogeneity; 25–50, modest heterogeneity; 50, high heterogeneity.

**Figure 3:**
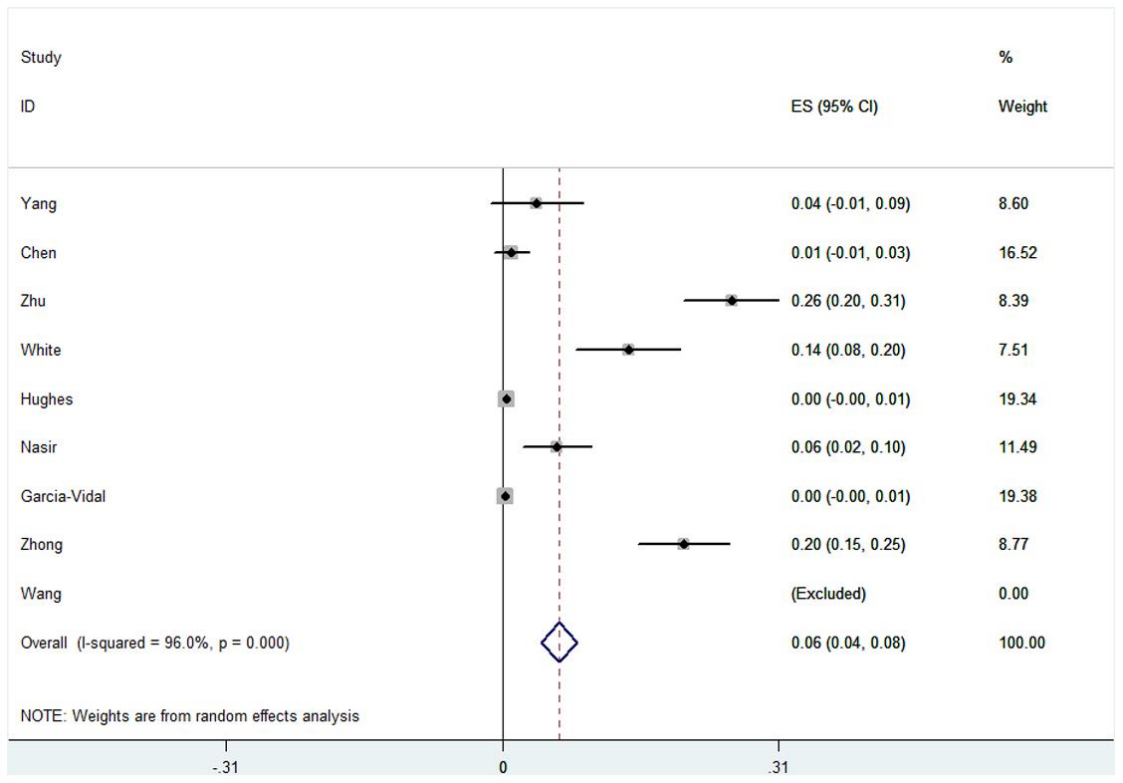
**Forest plot of aspergillus infection in COVID-19 patients.**

We also analyzed Aspergillus co-infection in COVID-19 patients, whereby the overall pooled proportion of patients was 0.06 (95% CI = 0.04-0.08, n = 2780, *I*^2^ = 96.0%). The difference in proportions between Asian and Caucasian patients was significant (0.13, 95% CI = 0.00-0.25, n = 673, *I*^2^ = 97.2% and 0.01, 95% CI = 0.00-0.03, n = 2107, *I*^2^ = 89.9% respectively). This correlation was not observed in other subgroups, such as race, study type or test method. However, we also failed to find a significantly different proportion between patients in ICU and the mixed hospitalized population (0.06 and 0.07). Specific data are summarized in Figure 3, Table 3 and Supplementary Figure 2.

**Table 3:** Proportion of Aspergillus co-infections and COVID-19.

	**Proportion(95%CI)**	***P***	***P_h_***	***I*^2^%**
**Total**	0.06(0.04-0.08)	0.000	0.000	96.0%
**Race**				
Asian	0.13(0.00-0.25)	0.046	0.000	97.2%
Caucasian	0.01(0.00-0.03)	0.028	0.000	89.9%
**Severity**				
ICU	0.06(-0.01-0.13)	0.111	0.000	88.5%
MIX	0.07(0.05-0.09)	0.000	0.000	97.4%
**Study type**				
Case series	0.01(-0.00-0.03)	0.147	0.320	0.0%
Retrospective study	0.13(0.01-0.25)	0.031	0.000	98.0%
Prospective study	0.07(-0.07-0.20)	0.318	0.000	95.3%
**Method**				
Culture	0.02(-0.00-0.04)	0.073	0.013	72.0%
RT-PCR	0.26(0.20-0.31)	0.000	-	-
MIX	0.07(-0.07-0.20)	0.314	0.000	95.2%
Fungus chip	0.20(0.15-0.25)	0.000	-	-
**Case size**				
>200	0.03(-0.00-0.07)	0.062	0.057	65.0%
<200	0.08(0.06-0.11)	0.000	0.000	97.6%

Mix of Severity, Symptoms of the disease include moderate, severe and critical; Mix of the Method, Detection methods include culture, serological diagnosis, and reverse transcription-polymerase chain reaction (RT-PCR); ICU, intensive-care unit; CI, confidence interval; *P*_h_, *P* value of heterogeneity, *P* value of Q-test for the heterogeneity test; *I*^2^, 0–25, no heterogeneity; 25–50, modest heterogeneity; 50, high heterogeneity.

**Figure 4:**
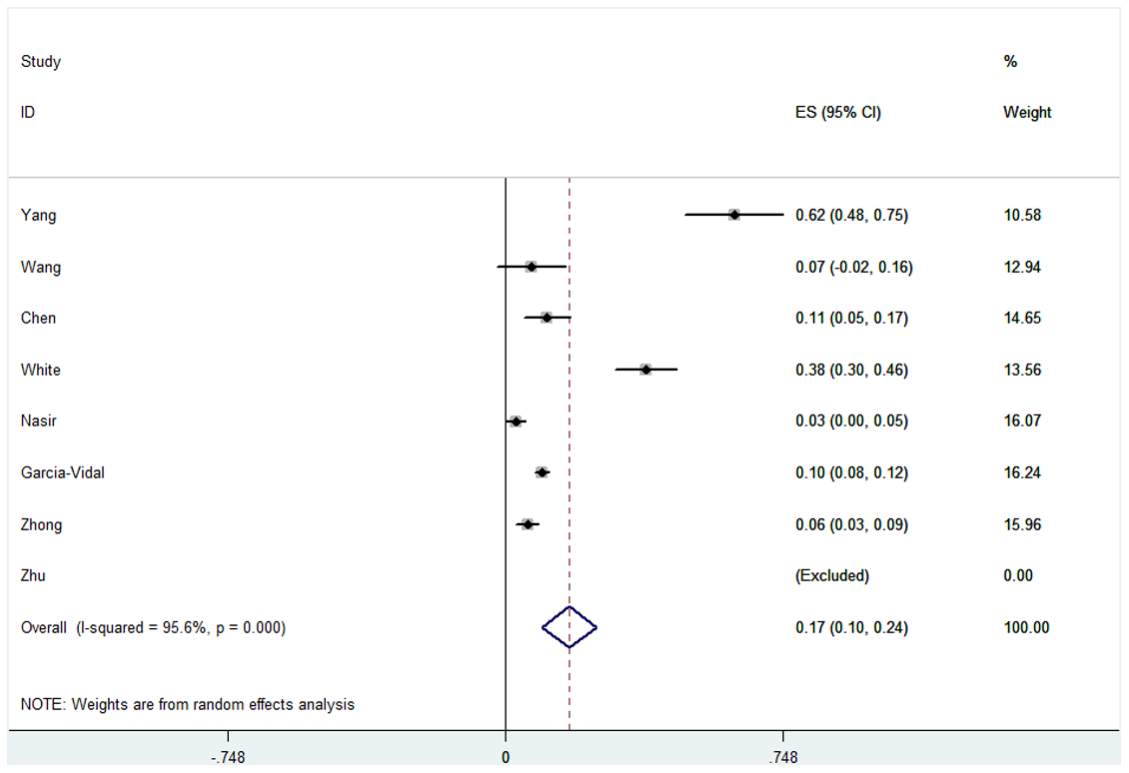
**Forest plot of mortality in COVID-19 patients with fungal co-infections.**

In terms of patient mortality with COVID-19 combined with fungal infection, we found that the overall pooled proportion of mortality was 0.17 (95% CI = 0.10-0.24, n = 1944, *I*^2^ = 95.6%). Subgroup analysis shown a significant difference in the proportions between patients in ICU and the mixed hospitalized population (0.36, 95% CI = 0.09-0.63, n = 286, *I*^2^ = 95.4% and 0.06 95% CI = 0.02-0.10, n = 1658, *I*^2^ = 84.6% respectively). We also found significantly different proportions in the case size of the subgroups (proportion in case size of the <200 subgroup was 0.23, 95% CI = 0.07-0.40, n = 462, *I*^2^ = 96.9% and proportion in case size of the >200 subgroup was 0.08, 95% CI = 0.04-0.12, n = 1482, *I*^2^ = 78.3%). This correlation was not observed in other subgroups, such as race, study type, test method, rate of Aspergillus and rate of fungal infection. Specific data are described in Figure 4, Table 4 and Supplementary Figure 3.

**Table 4:** Proportion of mortality in fungal co-infections and COVID-19.

	**Proportion(95%CI)**	***P***	***P_h_***	***I*^2^%**
**Total**	0.17(0.10-0.24)	0.000	0.000	95.6%
**Race**				
Asian	0.20(0.05-0.36)	0.012	0.000	95.4%
Caucasian	0.16(0.05-0.26)	0.004	0.000	97.1%
**Severity**				
ICU	0.36(0.09-0.63)	0.008	0.000	96.5%
MIX	0.06(0.02-0.10)	0.002	0.000	84.6%
**Study type**				
Case series	0.26(-0.01-0.53)	0.058	0.000	96.1%
Retrospective study	0.04(0.01-0.07)	0.008	0.116	59.6%
Prospective study	0.23(-0.04-0.51)	0.093	0.000	97.7%
**Method**				
Culture	0.16(0.07-0.24)	0.000	0.000	95.2%
MIX	0.38(0.30-0.46)	0.000	-	-
Fungus chip	0.06(0.03-0.09)	0.000	-	-
**Case size**				
>200	0.08(0.04-0.12)	0.000	0.032	78.3%
<200	0.23(0.07-0.40)	0.006	0.000	9.9%
**Rate of Aspergillus**				
<5%	0.18(0.08-0.28)	0.000	0.000	96.4%
>5%	0.17(-0.03-0.36)	0.093	0.000	96.1%
**Rate of fungal**				
<10%	0.16(0.07-0.24)	0.006	0.000	95.2%
>10%	0.20(-0.10-0.53)	0.175	0.000	98.0%

Mix of Severity, Symptoms of the disease include moderate, severe and critical; Mix of the Method, Detection methods include culture, serological diagnosis, and, reverse transcription-polymerase chain reaction; (RT-PCR), ICU, intensive-care unit; CI, confidence interval; *P*_h_, *P* value of heterogeneity, *P* value of Q-test for the heterogeneity test; *I*^2^, 0–25, no heterogeneity; 25–50, modest heterogeneity; 50, high heterogeneity.

### Publication bias and sensitivity analysis

Begg’s funnel plot and Egger’s test were performed to assess publication bias. We additionally conducted sensitivity analyses by omitting one study at a time in the calculation of a summary outcome (Supplementary Figures 1–3). Although the sample sizes for cases in all eligible studies varied, corresponding pooled proportions and 95% CIs were not qualitatively altered regardless of the study size. No other single study influenced pooled proportion and 95% CI qualitatively.

## DISCUSSION

The World Health Organization (WHO) declared COVID-19 as a public health emergency of international concern in February 2020 and since then, it has developed into a worldwide pandemic associated with substantial morbidity and mortality. At the time of writing, over 99 million laboratory-diagnosed cases of COVID-19 infections have been reported in 212 countries, contributing to over 2,100,000 death.

Antimicrobial therapy has a role in the treatment of suspected or confirmed bacterial or fungal respiratory co-infection [13]. This may be empirical or targeted in patients presenting at hospital, or for the management of nosocomial infection acquired during admission to hospital, such as hospital-acquired pneumonia or ventilator-associated pneumonia [14]. Patients may also be suffering from secondary co-infections not linked to their respiratory presentation, for example urinary tract or blood infections. Therefore, empirical treatment with antimicrobials is reasonable for bacterial/fungal pneumonia in unwell patients. Some national guidelines and evidence from cases have suggested the use of broad-spectrum antibiotics or the benefit of typical antibiotic cover.

Early studies have reported that patients with severe viral infection tend to get infections with Aspergillus, Candida, Cryptococcus neoformans, Pneumocystis, or other fungal species, and that this has an apparent association with increased morbidity and mortality [15–18]. Chen et al. [19] firstly described fungal infection in COVID-19 patients whereby the incidence was 4%, higher than that seen for bacterial infections, at only 1%. Since then, several studies have investigated the prevalence of fungal infections in patients with COVID-19. There are also an increasing number of reports from Europe, where patients with COVID-19 were associated with pulmonary aspergillosis [5, 11, 13]. In patients with acute respiratory distress syndrome caused by COVID-19, an increased risk of microbial infections has been reported, even in the absence of predisposing immunocompromising conditions. Epidemiological and clinical characteristics have been described in earlier research and some researchers have suggested that patients should be routinely screened for bacterial and fungal infection after a confirmation of COVID-19 infection [10, 11].

In this study, the overall pooled proportion of fungal infections in COVID-19 patients was 0.12, 95% CI = 0.07-0.16, *I*^2^ = 96.8%. Subgroup analysis of studies with separate data for Asian patients, showed that a greater proportion of them had fungal co-infections when compared to patients from non-Asian studies (0.15 versus 0.07). We also analyzed Aspergillus co-infection in COVID-19 patients and the overall pooled proportion of patients was 0.06 (95% CI = 0.04-0.08, *I*^2^ = 96.0%). Results of subgroup analysis were similar to fungal infection with COVID-19 patients. A likely explanation is that many physicians did not associate the combination of COVID-19 and fungal infection in the early stages of COVID-19, due to the urgency of the epidemic, which is an important reason for the confounding factors associated with patient illness. We suggest that all physicians should be more attentive to mycological diagnoses in COVID-19 patients at early stages of infection, to reduce the risk of this progressing to a critical illness.

In terms of mortality of COVID-19 patients combined with fungal infection, we found that the overall pooled proportion of mortality was 0.17 (95% CI = 0.10-0.24, *I*^2^ = 95.6%). Subgroup analysis showed a significant difference in proportions between patients in ICU and the mixed hospitalized population (0.36 versus 0.06). We also found significantly different proportions in the case size subgroup (proportion in case size of <200 subgroup was 0.23, 95% CI = 0.07-0.40, *I*^2^ = 96.9% and proportion in case size of >200 subgroup was 0.08, 95% CI = 0.04-0.12, *I*^2^ = 78.3%). This correlation was not observed in the rate of Aspergillus subgroup or rate of fungal subgroup. A potential explanation for this is that several physicians may not think that mycology detection is necessary for many terminally ill patients. In addition, mycological detection is also not validated for upper respiratory tract specimens in COVID-19 patients. In light of the current diagnostic difficulties and uncertainties relating to the risks associated with fungal infection in COVID-19 pneumonia, clinicians should maintain a high level of diligence for this infection in critically ill patients.

Some limitations of the current study need to be addressed [20]. First, only nine studies were examined, and the relatively small total sample size had limited power for the exploration of real associations. Second, subgroup analyses involved relatively small groups, which may not impart sufficient statistical power to explore the real association and are more likely to reveal greater beneficial effects than large-scale trials. Third, every physician has a different treatment for clinical diagnostic and treatment algorithms, which would allow for adjustments by other factors. In addition, inclusion of zero-event trials can sometimes decrease the effect size estimate and narrow confidence intervals.

## CONCLUSIONS

To the best of our knowledge, this is the only systematic review and meta-analysis investigating the proportions of fungal infection of COVID-19 patients within a large sample size. We summarized all available studies for an overall pooled proportion of fungal co-infection in COVID-19 patients. Overall, these findings provide evidence favoring a thoughtful empirical administration of antibiotics for the vast majority of patients with COVID-19 infection. Our analysis of the effects of utilizing antifungal therapy to treat COVID-19 patients can serve as a comprehensive reference for future COVID-19 treatment.

## MATERIALS AND METHODS

### Study selection

We searched Medline, Embase, MedRxiv and Cochrane Library with the search terms co-infection, coronavirus, severe acute respiratory syndrome coronavirus 2, SARS-CoV-2, 2019-nCoV and COVID-19 for studies published from January 1, 2020 up to December 1, 2020, and we manually searched the references of the selected articles for additional relevant articles (Figure 1). As this study was a meta-analysis, it did not require ethical approval.

### Data extraction and verification

Information pertaining to the enrolled studies is listed in Table 1, including: (I) the author’s name, (II) country or region of origin, (III) ethnicity or race, (IV) severity of illness, (V) study type, (VI) method, (VII) number of total patients, (VIII) number of patients in ICU, (IX) number of deaths, (X) fungal co-infection and (XI) Aspergillus co-infection. First, three authors (Jingwen Peng, Qiong Wang and Huan Mei) independently screened the citations for articles meeting our inclusion criteria and extracted all of the data. If at least two of them agreed, the study was included in the meta-analysis. The data included in the study were presented in the form of lists and differences were considered and identified in the quality evaluation. Next, everyone extracted while the other cross-checked the data. Disagreements were resolved by reviewing and discussion.

### Statistical analyses

The statistical significance of the pooled proportion was determined with the Z-test, and *P*-values < 0.05 were considered as statistically significant. Data were pooled from the meta-analysis with the random-effects model using the DerSimonian and Laird method, and the fixed-effects model using the Mantel–Haenszel method. To evaluate the influence of individual data sets by overall pooled proportion, we conducted forest plot analysis to determine the stability of our results. We also carried out sensitivity analysis in which a single study within the overall meta-analysis was deleted one at a time. We applied Funnel plots and Egger’s linear regression test to assess publication bias [20]. All statistical analyses were carried out using STATA version 11.0 (Stata Corporation College Station, TX, USA).

## Supplementary Material

Supplementary Figures
