# Supplementary material for: Fungal co-infection in COVID-19 patients: evidence from a systematic review and meta-analysis
Source: Aging (Albany NY). 2021 Mar 19;13(6):7745–57. doi: 10.18632/aging.202742 (PMC8034918; doi:10.18632/aging.202742)
Supplement: Supplementary Figures [file aging-13-202742-s001.pdf]

SUPPLEMENTARY FIGURES

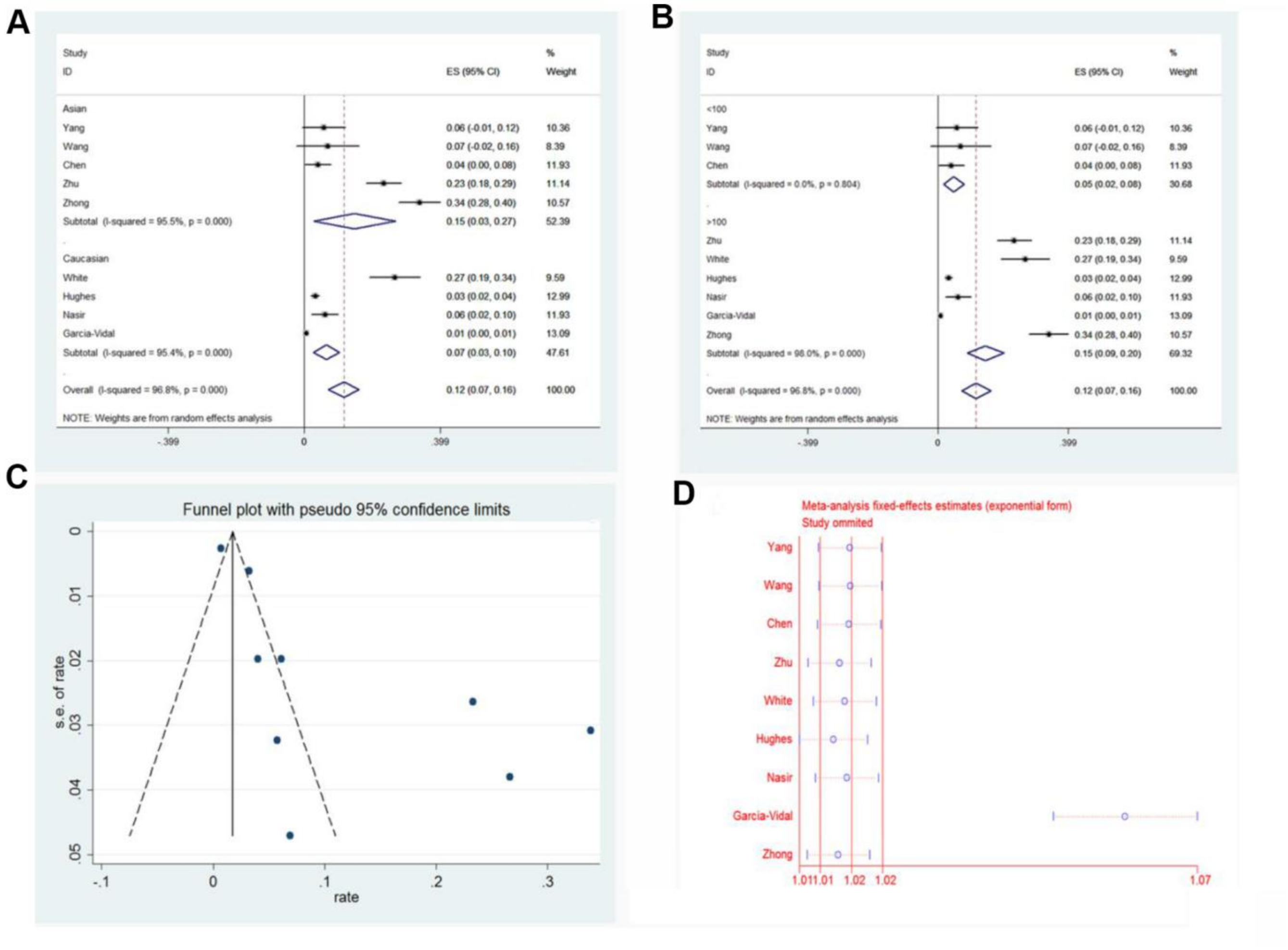

Supplementary Figure 1. The association of fungal infection in COVID-19 patients. (**A**, **B**) Forest plot of fungal infection in COVID-19 patients (**A**) Race subgroup analysis (**B**) Case size subgroup analysis (**C**) The funnel plot of fungal infection in COVID-19 patients (**D**) Sensitivity analysis of fungal infection in COVID-19 patients.

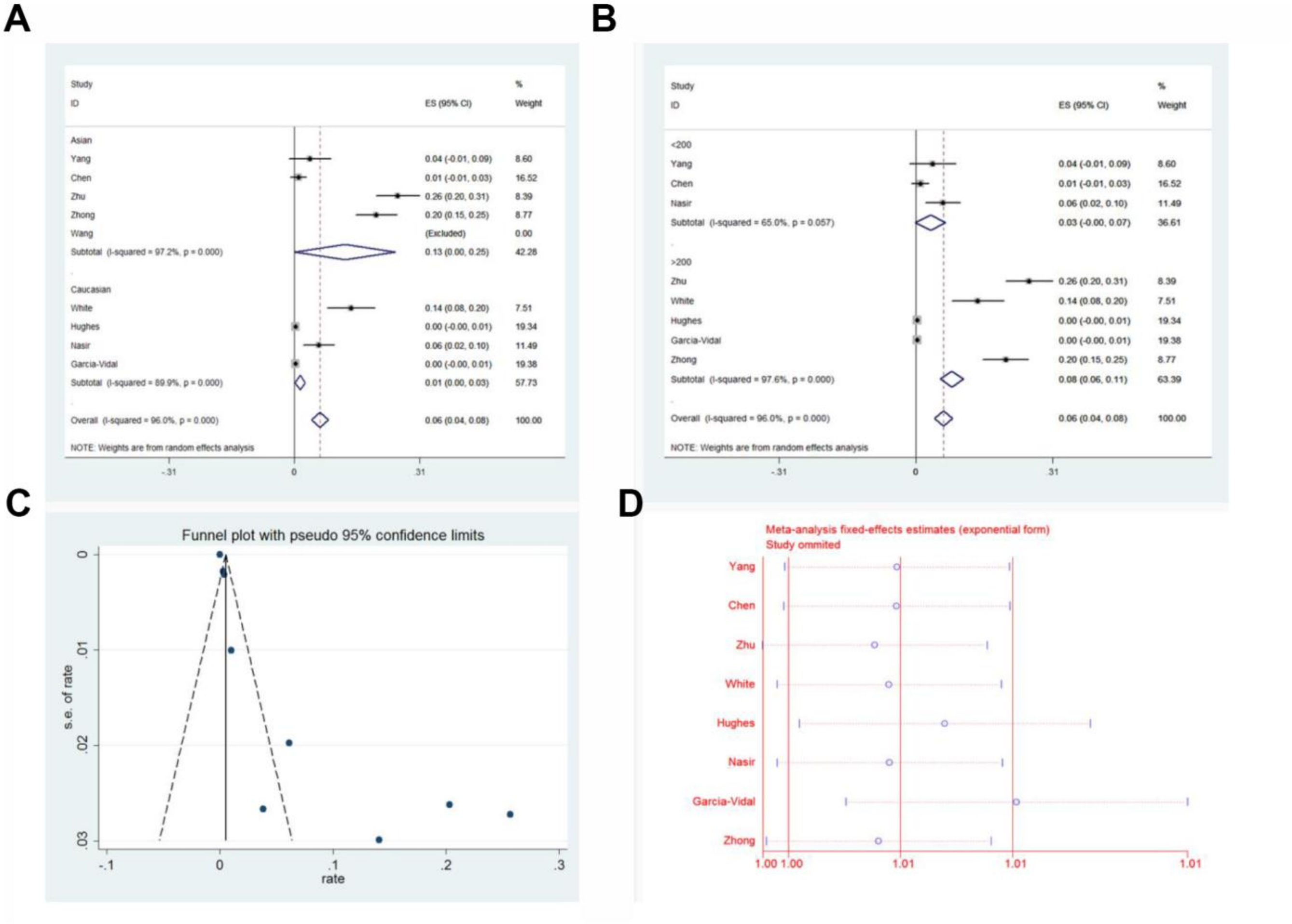

Supplementary Figure 2. The association of aspergillus infection in COVID-19 patients. (**A**, **B**) Forest plot of aspergillus infection in COVID-19 patients (**A**) Race subgroup analysis (**B**) Case size subgroup analysis (**C**) The funnel plot of aspergillus infection in COVID-19 patients (**D**) Sensitivity analysis of aspergillus infection in COVID-19 patients.

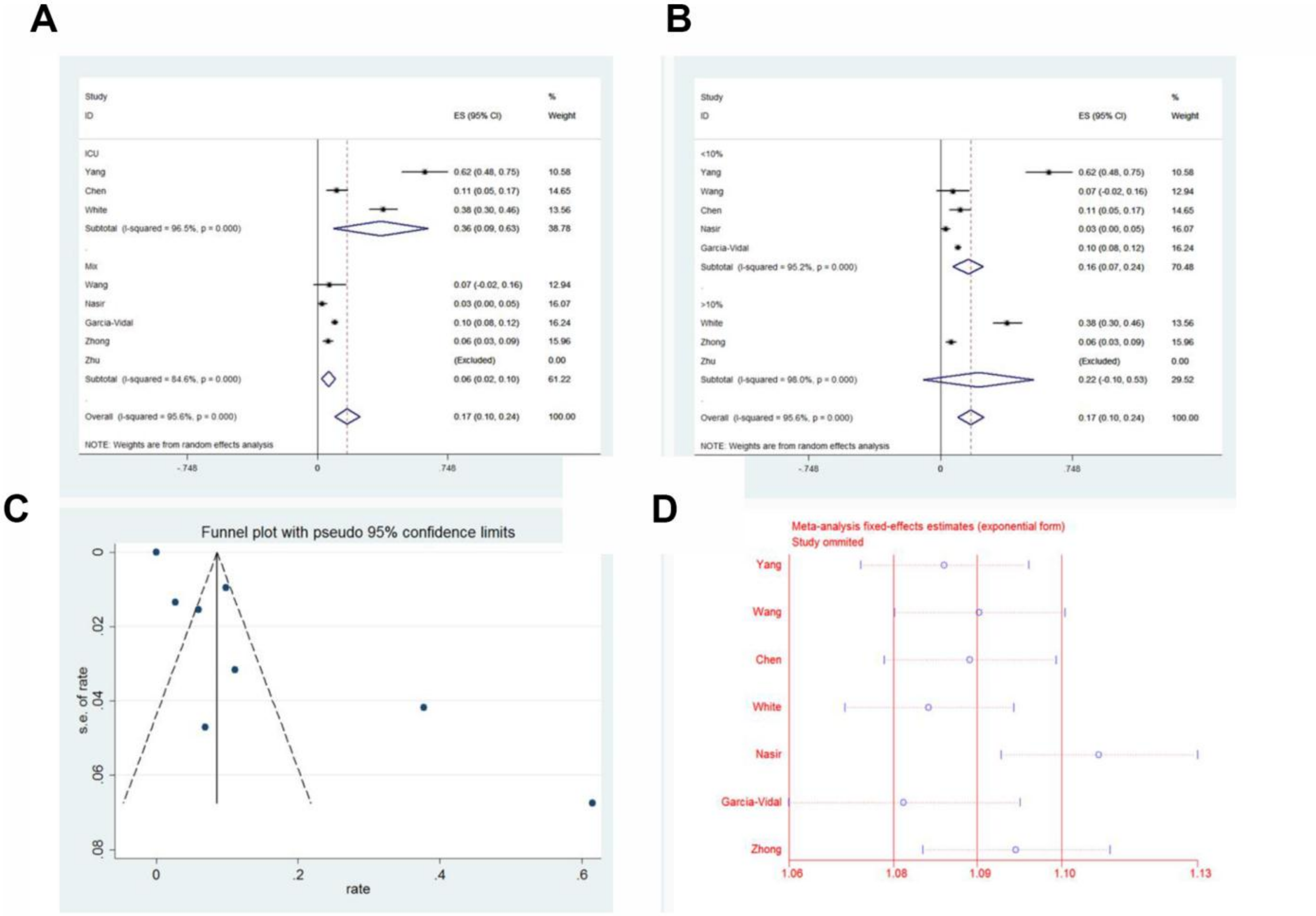

Supplementary Figure 3. The association of mortality in fungal co-infections and COVID-19 patients. (**A**, **B**) Forest plot of mortality in fungal co-infections and COVID-19 patients (**A**) Severity subgroup analysis (**B**) Case size subgroup analysis (**C**) The funnel plot of mortality in fungal co-infections and COVID-19 patients (**D**) Sensitivity analysis of mortality in fungal co-infections and COVID-19 patients.
